# A TMO-ZnO Heterojunction-Based Sensor for Transformer Defect Detection: A DFT Study

**DOI:** 10.3390/nano15110856

**Published:** 2025-06-03

**Authors:** Jingyi Yan, Weiju Dai, Dexu Zou, Haoruo Sun, Chao Tang, Yingang Gui

**Affiliations:** 1 Electric Power Research Institute of Yunnan Power Grid, Kunming 650214, China; yanjingyi1998@163.com (J.Y.); 15911530479@163.com (W.D.); zdxdb@126.com (D.Z.); s_hr1997@163.com (H.S.); 2 College of Engineering and Technology, Southwest University, Chongqing 400716, China; swutc@swu.edu.cn

**Keywords:** metal oxide heterojunction, dissolved gases, density functional theory, transformer defect detection, energy efficiency analysis

## Abstract

The gas adsorption and sensing properties of a transition metal oxide (TMO)-ZnO heterojunction-based sensor for H_2_, CO, and C_2_H_4_ are analyzed. It is found that CuO, Ag_2_O, and Cu_2_O stably composite onto the surface of ZnO by forming heterojunctions, which helps to improve the gas sensing and selectivity of the sensor. The adsorption results show that CuO-ZnO shows physical adsorption for H_2_ and good gas sensing performance for CO and C_2_H_4_, while Ag_2_O-ZnO and Cu_2_O-ZnO have significant responses for H_2_, CO, and C_2_H_4_. In addition, the introduction of the TMO-ZnO heterojunction structure can effectively avoid the sensor poisoning phenomenon, as the gas adsorption process does not destroy the original geometric configuration of the heterojunction. This study lays a theoretical foundation for preparing TMO-ZnO heterojunction-based sensors for transformer defect detection and energy efficiency analysis.

## 1. Introduction

Oil-immersed transformers are crucial components that are widely used in power systems [1,2]. With the sharp increase in social electricity consumption, the power system has put forward higher energy efficiency requirements for these numerous transformers [3]. The energy efficiency of transformers is not only affected by the materials (silicon steel, amorphous materials), structure (stacked and rolled core), and production process [4], but also by complex transformer defects during long-term operation, such as impulse discharge, short circuiting between winding, overheating, etc. [5]. Therefore, it is necessary to track and monitor the running status and energy efficiency level of transformers in operation and to gradually eliminate old transformers that do not meet the energy efficiency standards. Studies have shown that dissolved gas analysis in transformer oil has become a convenient and feasible method for monitoring the operating status of transformers, diagnosing transformer defect types, and ensuring the high running efficiency of transformers [6,7].

Metal oxide semiconductor-based gas sensors have attracted widespread attention from researchers around the world due to their high sensitivity, low cost, and fast response time [8,9]. At present, metal oxide gas sensing materials that are extensively studied include ZnO, CuO, Ag_2_O, Cu_2_O, etc. [10,11,12,13]. However, single metal oxide gas sensors generally have drawbacks, such as operating temperature requirements, low detection sensitivity, low selectivity, and a long response time. However, when a heterojunction is formed between two different gas sensing materials, the charge transfer caused by the inconsistent Fermi energy levels of the two metal oxides forms a charge depletion layer and potential barrier at the interface of the two different metal oxide semiconductor materials [14]. Combined with the synergistic effect of the small size and high specific surface area of the metal oxide, the gas sensing response characteristics of the metal oxide heterojunction gas sensor are improved [15].

Li et al. successfully prepared a unique graded radial CeO_2_-ZnO heterostructure with excellent triethylamine gas sensing performance through a feasible solvent thermal method [16]. Yuan et al. proposed a new etching and calcination method to prepare porous ZnO-Co_3_O_4_ nanoplates, which exhibit excellent performance in CO detection [17]. Meng et al. successfully synthesized NiO-SnO_2_ heterojunction micropowders assembled from thin porous nanosheets through a simple one-step hydrothermal route [18]. The study showed that NiO-SnO_2_ micropowder sensors still exhibit high response characteristics in lower operating temperature regions. Meanwhile, Duoc et al. prepared a highly selective and responsive NO_2_ gas sensor based on a ZnO-SnO_2_ heterojunction [19]. Hsu et al. prepared CuO-ZnO heterojunction nanofibers that can be used as gas sensing materials. At the optimal operating temperature, their response to H_2_S is about 25.79% higher than that of pure ZnO sensors [20].

Based on density functional theory (DFT), this study proposes a metal oxide heterojunction-based gas sensor for dissolved gas in transformer oil detection. The gas sensing mechanism of the metal oxide heterojunction-based gas sensor for H_2_, CO, and C_2_H_4_ has been explored by analyzing the heterojunction model, adsorption structure, adsorption energy, adsorption distance, energy band, charge transfer, density of states (DOS), differential charge density (DCD), and molecular orbitals. This study provides a theoretical foundation for preparing a specific gas sensor for use in transformer defect detection and energy efficiency analysis.

## 2. Methods

All theoretical calculations are performed based on DFT calculations [21]. The Hirshfeld method was used for atomic charge analysis. The Perdew–Burke–Ernzerhof (PBE) function from the generalized gradient approximation (GGA) was selected to calculate the electron exchange energy and correlation energy [22]. The orbital electrons were calculated using the DFT pseudopotentials method. The Tkatchenko and Scheffler (TS) algorithm was chosen to correct the van der Waals force for more accurate results [23]. The convergence criteria were set according to previous studies [24,25,26]. A 5 × 5 × 1 k point sampling of the Monkhorst Pack scheme to perform was used to deal with the Brillouin zone [27,28]. The supercell model was established by expanding the original cell so that the sizes of the two semiconductors were close to each other to reduce the lattice mismatch. The DFT method was used to calculate the adsorption characteristics of the heterojunction for the dissolved gas in the transformer and the gas sensing potential. We have searched the literature for studies with the same research method in this paper to ensure the consistency of the calculation results with other simulation studies [29,30]. However, there are still some differences between the simulation and experimental data [31,32]. The main reason for this is that the characteristics of the material are affected by the crystal plane.

The formation energy (*E_form_*) of heterojunctions formed by two types of metal oxide semiconductors is defined as Equation (1), where *E_Heterojunction_*, *E_above_*, and *E_below_* represent the total energy of the heterostructure, the energy of the upper metal oxide, and the energy of the lower metal oxide, respectively. The adsorption energy (*E_ads_*) of target gas molecules on a heterojunction model is defined as Equation (2), where *E_gas/Heterojunction_*, *E_Heterojection_*, and *E_gas_* represent the total energy of gas molecules adsorbed by the heterojunction model, the total energy of the heterojunction structure, and the energy of the gas molecules, respectively. The charge transfer (*Q_t_*) between gas molecules and heterojunctions is defined as Equation (3), where *Q_a_* and *Q_b_*, respectively, represent the net charges carried by gas molecules after adsorption and the net charges carried by gas molecules themselves before adsorption. Based on the frontier orbital theory, the energy gap (*E_g_*) of molecular orbitals is defined as Equation (4), where *E_LUMO_* and *E_HOMO_* represent the energy of the lowest unoccupied molecular orbital (LUMO) and the highest occupied molecular orbital (HOMO) of the entire heterojunction, respectively. The relationship between *Eg* and electronic conductivity (*σ*) can be expressed by Equation (5), where *K_B_* is the Boltzmann constant (8.62 × 10^−5^ eV/K) and *T* denotes temperature. It can be seen from Equation (5) that when *Eg* decreases, the electron conductivity will increase accordingly, and vice versa. Equations (1)–(5) are as follows:

*E_form_* = *E_Heterojunction_* − *E_above_* − *E_below_* (1)

*E_ads_* = *E_gas/Heterojunction_* − *E_Heterojunction_* − *E_gas_* (2)

*Q_t_* = *Q_a_* − *Q_b_* (3)

*E_g_* = |*E_LUMO_* − *E_HOMO_*| (4)

*σ*∝e^(*−Eg/2KBT*)^ (5)

## 3. Results and Discussion

### 3.1. Gas Sensing Properties of the CuO-ZnO Heterojunction for H_2_, CO, and C_2_H_4_

#### 3.1.1. Structure and Electronic Analysis of the CuO-ZnO Heterojunction

**Figure 1:**
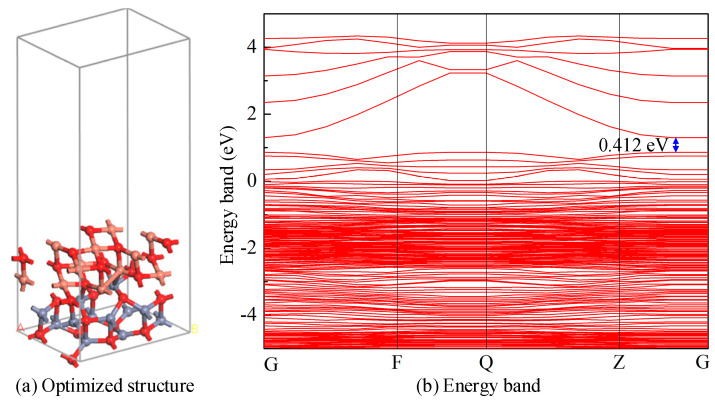
(**a**) Optimized structure of the CuO-ZnO heterojunction. (**b**) Energy band of CuO-ZnO heterojunction.

A 3 × 2 × 1 CuO-ZnO two-layer heterojunction was constructed, where CuO serves as the upper layer metal oxide, while ZnO serves as the lower layer-based metal oxide. The lattice mismatch of the CuO-ZnO heterojunction is within a 5%. Geometric optimization is performed on the constructed CuO-ZnO heterojunction model, and the most stable structure is obtained after convergence, as shown in Figure 1a. The lattice constants of the CuO-ZnO heterojunction are *a* = 9.5217 Å, *b* = 6.3451 Å, and *c* = 28.9218 Å; *γ* = 117.8359°, containing 48 atoms. Multiple chemical bonds form between CuO and ZnO metal oxides, with bond lengths ranging from 2.088 Å to 2.706 Å. Meanwhile, the formation energy of the CuO-ZnO heterojunction model is calculated to be −3.513 eV. The energy band of the optimized CuO-ZnO metal oxide heterojunction is shown in Figure 1b. It can be observed that the bandgap width of the CuO-ZnO heterojunction is 0.412 eV. Compared with the bandgap of CuO and ZnO, the energy band of the CuO-ZnO heterojunction becomes significantly denser, and the bandgap width is significantly narrower than that of CuO and ZnO. Therefore, the conductivity of the CuO and ZnO heterojunction increases significantly.

#### 3.1.2. Structure and DCD Analysis of the Gas/CuO-ZnO Heterojunction System

**Figure 2:**
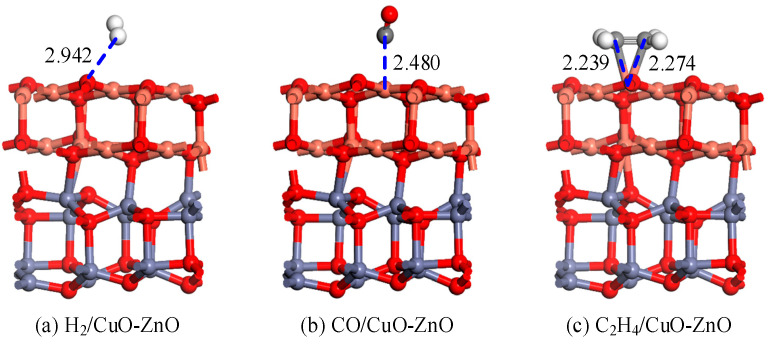
Structural models of H_2_, CO, and C_2_H_4_ adsorption on the CuO-ZnO heterojunction. The distance is in Å.

To obtain the most stable gas adsorption structure, the geometric optimization of the established model was carried out by considering a variety of H_2_, CO, and C_2_H_4_ gas molecules approaching the CuO-ZnO heterojunction. Models with horizontal proximity, vertical proximity, and different angles of inclination were considered. When searching for the most stable adsorption models of CO and C_2_H_4_, the models of different atomic proximities were also considered. Figure 2 shows the most stable adsorption structure of gas molecules adsorbed on CuO-ZnO heterojunction. In the geometric optimization process involving molecular adsorption, the entire heterostructure was relaxed. For comparison, the adsorption parameters of CuO, ZnO, and CuO-ZnO heterojunction for H_2_, CO, and C_2_H_4_ were calculated, as shown in Table 1. The adsorption energies of the CuO-ZnO heterojunction for H_2_, CO, and C_2_H_4_ gas molecules are ranked as follows in descending order: C_2_H_4_ (−0.922 eV) > CO (−0.292 eV) > H_2_ (−0.161 eV). All adsorption energies were negative, indicating that the adsorption process is an exothermic reaction. Compared with the adsorption of the intrinsic metal oxides CuO and ZnO, the adsorption energy of the CuO-ZnO heterojunction for gas molecules increased to varying degrees compared to CuO and decreased to varying degrees compared to ZnO. For H_2_ adsorption, the H atom is close to the O atom on the surface of the CuO-ZnO heterojunction, with an adsorption distance of 2.942 Å. For CO adsorption, the C atom in the gas molecule is close to the Cu atom on the heterojunction surface, with an adsorption distance of 2.942 Å. For the adsorption of C_2_H_4_, both C atoms in the gas molecule are close to the surface Cu atoms and form two Cu-C bonds with bond lengths of 2.239 Å and 2.274 Å, respectively. Compared with intrinsic CuO adsorption, the heterojunctions have a certain degree of shortened adsorption distance for all three gas molecules. Compared with the intrinsic adsorption of ZnO, the heterojunction not only shortens the adsorption distance of C_2_H_4_, but also increases the adsorption distance of the other two gases to a certain extent. Meanwhile, there is no significant change in the structure of the heterojunction before and after gas molecule adsorption, indicating that the CuO-ZnO heterojunction has strong chemical stability.

**Figure 3:**
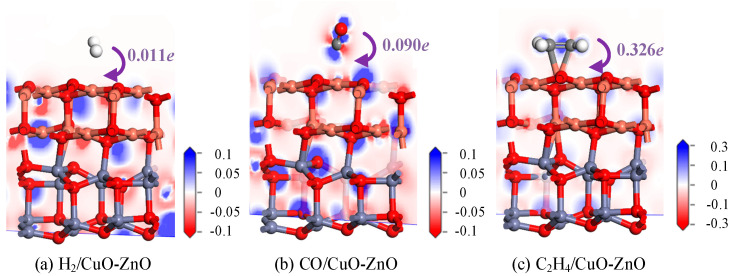
DCD diagrams of H_2_-, CO-, and C_2_H_4_-adsorbed CuO-ZnO heterojunctions.

To analyze the electron transfer during the adsorption process of gas molecules, the DCD of H_2_-, CO-, and C_2_H_4_-adsorbed CuO-ZnO heterojunction systems were calculated. The corresponding DCDs of the three adsorption systems are shown in Figure 3. The red area in the figure indicates a decrease in electron density, while the blue area indicates an increase in electron density. As shown in Figure 3a, after the CuO-ZnO heterojunction adsorbs H_2_, there is no significant color change around the gas molecules. H_2_ only transferred 0.011 *e* of electrons to the surface of the heterojunction, indicating that the charge transfer phenomenon between the gas molecules and the heterojunction surface is very weak during the adsorption process. As shown in Figure 3b, the blue color around the O atom after CO adsorption on the surface indicates that it has obtained electrons, thereby increasing the electron density in this region. On the contrary, the appearance of red around the C atom indicates that it provides electrons as an electron donor, thereby reducing the electron density in that region. The adsorption results in the transfer of 0.090 *e* electrons from CO to the heterojunction surface. Similarly, as shown in Figure 3c, the C atom in C_2_H_4_ is surrounded by blue to show that it is obtaining electrons, while the H atom is surrounded by red to indicate that it is losing electrons. Charge distribution analysis shows that C_2_H_4_ transferred 0.326 *e* electrons to the heterojunction surface. From the above DCD analysis, it can be observed that the two Cu and Zn metal atoms of the CuO-ZnO heterojunction are always surrounded by a red color, indicating that they always act as electron donors, while the O atom is always surrounded by a blue color, indicating that it always acts as an electron acceptor. From the adsorption structure and DCD analysis discussed above, it can be seen that H_2_ has a long adsorption distance, low adsorption energy, and low electron transfer on the CuO-ZnO heterojunction, indicating a weak physical adsorption. For the adsorption of C_2_H_4_, the formation of chemical bonds, high adsorption energy, and intense electron transfer phenomena indicate that it is a highly interacting chemical adsorption.

#### 3.1.3. DOS and Molecular Orbital Analysis of the Gas/CuO-ZnO Heterojunction System

**Figure 4:**
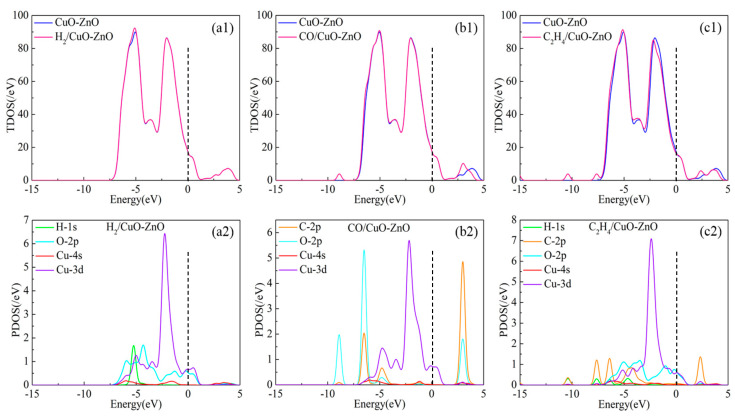
TDOS and PDOS of the H_2_-, CO-, and C_2_H_4_-adsorbed CuO-ZnO heterojunction. (**a1**) TDOS of H_2_-adsorbed CuO-ZnO heterojunction; (**a2**) PDOS of H_2_-adsorbed CuO-ZnO heterojunction; (**b1**) TDOS of CO-adsorbed CuO-ZnO heterojunction; (**b2**) PDOS of CO-adsorbed CuO-ZnO heterojunction; (**c1**) TDOS of C_2_H_4_-adsorbed CuO-ZnO heterojunction; (**c2**) PDOS of C_2_H_4_-adsorbed CuO-ZnO heterojunction.

To further investigate the adsorption mechanism of CuO-ZnO heterojunctions on different target gases, the total density of states (TDOS) and partial density of states (PDOS) of all adsorption systems were analyzed, and the corresponding results are shown in Figure 4. The position of the Fermi level is near the dotted line in Figure 4. By observing the changes in the TDOS of the adsorption system near the Fermi level, the influence of gas adsorption on the conductivity of heterojunctions can be determined. It can be observed that there is no significant change in the TDOS of the adsorption system after H_2_ adsorbs on the surface of the heterojunction compared with that before gas adsorption, indicating that the influence of H_2_ on the conductivity of the CuO-ZnO heterojunction is minimal. For CO adsorption, the TDOS of the adsorption system near the Fermi level decreases, while it shows a significant increase for C_2_H_4_ adsorption, reflecting a certain degree of increase in the conductivity of the former adsorption system and a significant decrease in the conductivity of the latter. The reason for the changes in TDOS is due to the redistribution of electrons caused by the interaction between gas molecules and heterojunctions during the adsorption process. For H_2_ adsorption, the hybridization phenomenon between atomic orbitals is weak, and mainly occurs near the −5 eV level that far from the Fermi level. Therefore, its impact on TDOS and conductivity is minimal. Upon CO adsorption, the peaks of the atomic orbitals Cu-4*s* and Cu-3*d* of the C-2*p*, O-2*p*, and Cu atoms overlap at the −7 eV, −5 eV, −1 eV, and 3 eV energy levels, respectively, resulting in changes in the TDOS and affecting conductivity. For the adsorption of C_2_H_4_, the PDOS of the adsorption system shows that strong orbital hybridization occurs between atomic orbitals in the energy level range of −8 eV to 3 eV. The strong atomic interaction corresponds to the formation of Cu-C chemical bonds in the system, causing significant changes in the corresponding TDOS.

**Figure 5:**
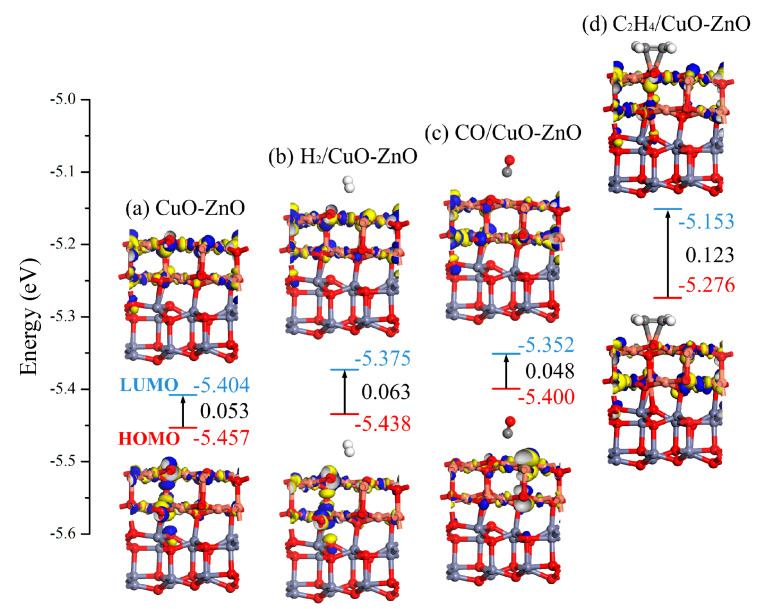
HOMO and LUMO distribution before and after gas adsorption on the CuO-ZnO heterojunction.

From a molecular orbital perspective, the interaction mechanism between gas molecules and heterojunction surfaces was studied, and the changes in system conductivity during the reaction process were evaluated. Figure 5 shows the distribution of conductivity and LUMO before and after gas adsorption on the CuO-ZnO heterojunction. The corresponding values of *E_LUMO_*, *E_HOMO_*, and *E_g_* for each system are marked in the figure. It can be observed that the adsorption systems of CO and C_2_H_4_ lead to a significant change in the distribution of HOMO and LUMO compared to the CuO-ZnO heterojunction without gas adsorption. Meanwhile, it can be observed that H_2_ has little effect on the distribution of the heterojunction’s HOMO and LUMO. Without gas adsorption, the *E_g_* of the CuO-ZnO heterojunction is 0.053 eV. Compared with the system after gas adsorption, except for the CO adsorption system where the *E_g_* value decreases to 0.048 eV, the *E_g_* values of H_2_ and C_2_H_4_ adsorption systems increase. Especially for the adsorption of C_2_H_4_, the *E_g_* value of the adsorption system increases to 0.123 eV, reflecting a certain degree of decrease in conductivity. The above molecular orbital analysis is consistent with the DOS analysis results.

### 3.2. Gas Sensing Properties of the Ag_2_O-ZnO Heterojunction for H_2_, CO, and C_2_H_4_

#### 3.2.1. Structure and Electronic Analysis of Ag_2_O-ZnO Heterojunction

**Figure 6:**
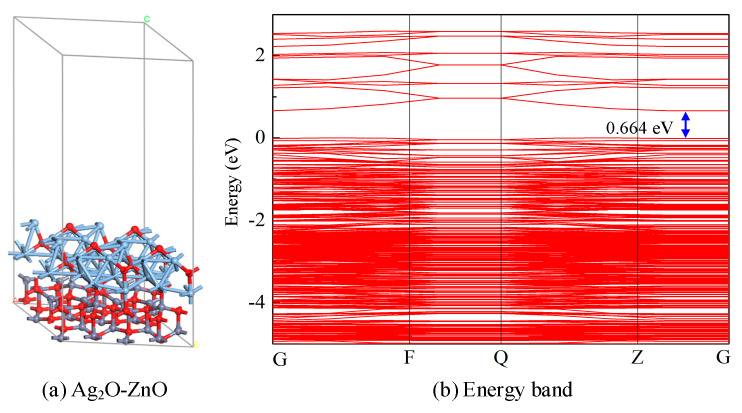
(**a**) Optimized structure of the Ag_2_O-ZnO heterojunction. (**b**) Energy band of the Ag_2_O-ZnO heterojunction.

A 3 × 3 × 1 Ag_2_O-ZnO two-layer heterojunction was constructed. For building the Ag_2_O-ZnO heterojunction structural model, Ag_2_O sites were used as the upper layer metal oxide, and ZnO sites were used as the lower layer-based metal oxide. The lattice mismatch of the Ag_2_O-ZnO heterojunction is within 5%. Geometric optimization was performed on the constructed Ag_2_O-ZnO heterojunction model, and the most stable structure was obtained, as shown in Figure 6. The supercell of Ag_2_O-ZnO heterojunction is shown in Figure 6a, with corresponding lattice constants of *a* = 13.1737 Å, *b* = 13.1737 Å, and *c* = 31.1638 Å; *γ* = at 120.000°, which consists of a total of 112 atoms. From the structural diagram, it can be observed that the Ag_2_O-ZnO heterojunction forms a highly symmetrical regular geometric structure, and the numerous triangular structures formed by the upper layer of Ag_2_O are conducive to improving the structural stability of the model. Meanwhile, multiple O-C and Ag-O bonds form between two different metal oxides, namely Ag_2_O and ZnO, with chemical bond lengths ranging from 1.968 Å to 2.213 Å. The formation energy of the structural model is calculated to be −13.344 eV, and the formation energy is less than 0 eV, indicating that the heterojunction can exist stably. As shown in Figure 6b, the bandgap width of the optimized Ag_2_O-ZnO metal oxide heterojunction model is 0.664 eV. Compared with the bandgap of intrinsic Ag_2_O and ZnO metal oxides, the energy band of Ag_2_O-ZnO heterojunction becomes denser, and the bandgap width is shortened to a certain extent, indicating that the formation of this heterojunction improves the conductivity of the system.

#### 3.2.2. Structure and DCD Analysis of Gas/Ag_2_O-ZnO Heterojunction System

**Figure 7:**
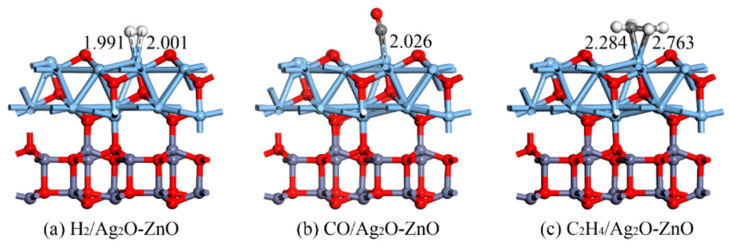
Structural models of H_2_, CO, and C_2_H_4_ adsorption on the Ag_2_O-ZnO heterojunction. The distance is in Å.

As in Section 3.1.2, the gas adsorption structures on the Ag_2_O-ZnO heterojunction were built and optimized by placing H_2_, CO, and C_2_H_4_ gas molecules in different directions and atomic proximity forms onto the surface of the Ag_2_O-ZnO heterojunction. The most stable adsorption structure of gas molecules adsorbed on the surface of the Ag_2_O-ZnO heterojunction is shown in Figure 7, and the corresponding adsorption parameters are shown in Table 2. In the geometric optimization process involving molecular adsorption, the entire heterostructure was relaxed. The magnitude of the adsorption energy to some extent reflects the strength of the system’s adsorption capacity. The adsorption capacity of the Ag_2_O-ZnO heterojunction for the H_2_, CO, and C_2_H_4_ gas molecules is ranked from strong to weak as follows: C_2_H_4_ (−1.300 eV) > CO (−1.083 eV) > H_2_ (−0.346 eV). Similarly, the adsorption distance of the heterojunctions for these three gas molecules exhibits a similar pattern to the adsorption energy, as follows: C_2_H_4_ (2.284 Å) > CO (2.026 Å) > H_2_ (1.991 Å). Compared with the intrinsic adsorption of Ag_2_O and ZnO, the adsorption distance of heterojunctions for gas molecules is shortened to a certain extent. For H_2_ adsorption, both H atoms in H_2_ form two Ag-H bonds with surface Ag atoms with bond lengths of 1.991 Å and 2.001 Å, respectively, resulting in a decrease in adsorption energy compared to Ag_2_O and ZnO. For CO adsorption, the C atom is close to the surface Ag atom and forms an Ag-C bond with a bond length of 2.026 Å. The adsorption energy is increased compared to the intrinsic substrate ZnO, and decreased compared to Ag_2_O. For the adsorption of C_2_H_4_, two C atoms form two Ag-C bonds with a surface Ag atom with bond lengths of 2.284 Å and 2.763 Å, respectively. The change in the heterojunction adsorption energy is similar to that of CO adsorption, with the adsorption energy smaller than Ag_2_O and greater than ZnO. At the same time, it can be observed that Ag_2_O-ZnO does not change its geometric structure for the adsorption of any gas molecule, indicating its strong structural stability.

**Figure 8:**
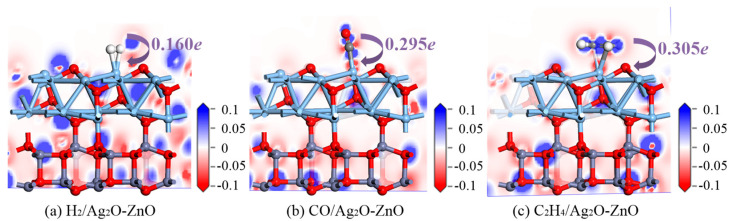
DCD diagrams of H_2_, CO, and C_2_H_4_ adsorbed Ag_2_O-ZnO heterojunction.

The DCDs of H_2_, CO, and C_2_H_4_ adsorption on the Ag_2_O-ZnO heterojunction were analyzed to explore the electron transfer during the adsorption process. The corresponding DCDs of the three adsorption systems are shown in Figure 8. Similarly, the red area indicates a decrease in electron density, while the blue area indicates an increase in electron density. As shown in Figure 8a, the DCD distribution of the H_2_-adsorbed Ag_2_O-ZnO heterojunction shows that the surrounding area of H_2_ is slightly reddish, indicating the transfer of electrons from gas molecules to the heterojunction surface. Charge distribution analysis of the electron transfer amount is calculated to be 0.160 *e*. The DCD diagram of CO adsorption is shown in Figure 8b. The C and O atoms in the gas molecule are surrounded by red and blue, respectively, indicating that the former loses electrons while the latter gains electrons. Overall, charge distribution analysis shows that electrons transfer from the gas molecule to the heterojunction, with a charge of 0.295 *e*. Figure 8c shows the adsorption of C_2_H_4_, with a red color near the four H atoms, while the two C atoms are surrounded by a blue color. Charge distribution analysis shows that C_2_H_4_ ultimately provides electrons with a charge of 0.305 *e* to the surface atoms of the heterojunction. Similarly, for Ag_2_O-ZnO heterojunctions, the O atom always acts as an electron acceptor, while the Ag and Zn metal atoms always act as electron donors. Based on the above analysis, it can be concluded that the adsorption of H_2_, CO, and C_2_H_4_ on the surface of the Ag_2_O-ZnO heterojunction is a highly reactive chemical adsorption according to the analysis of adsorption energy, adsorption distance, electron transfer, and formation of corresponding chemical bonds. The heterojunction has a high sensitivity to detecting these three gases.

#### 3.2.3. DOS and Molecular Orbital Analysis of Gas/Ag_2_O-ZnO Heterojunction System

**Figure 9:**
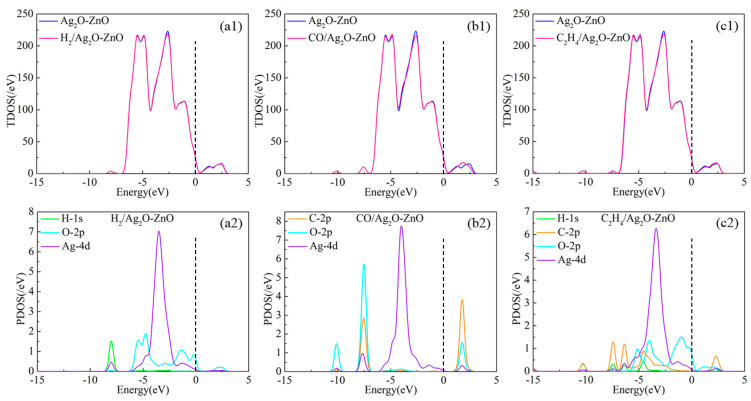
TDOS and PDOS of H_2_-, CO-, and C_2_H_4_-adsorbed Ag_2_O-ZnO heterojunctions. (**a1**) TDOS of H_2_-adsorbed Ag_2_O-ZnO heterojunction; (**a2**) PDOS of H_2_-adsorbed Ag_2_O-ZnO heterojunction; (**b1**) TDOS of CO-adsorbed Ag_2_O-ZnO heterojunction; (**b2**) PDOS of CO-adsorbed Ag_2_O-ZnO heterojunction; (**c1**) TDOS of C_2_H_4_-adsorbed Ag_2_O-ZnO heterojunction; (**c2**) PDOS of C_2_H_4_-adsorbed Ag_2_O-ZnO heterojunction.

By observing the TDOS of H_2_, CO, and C_2_H_4_ gas molecules adsorbed on the Ag_2_O-ZnO shown in Figure 9, the position of the Fermi level can be seen to be near the dotted line in Figure 9. It can be found that the TDOS of these three adsorption systems near the Fermi level has increased to varying degrees compared to Ag_2_O-ZnO without gas adsorption, indicating that the conductivity of the Ag_2_O-ZnO heterojunction has decreased after adsorbing these three gas molecules. For H_2_ adsorption, the peak overlap between O-2*p* and Ag-4*d* in the energy level range of −6 eV to 0 eV is the main reason affecting the changes in TDOSs. For CO adsorption, the peaks of C-2*p*, O-2*p*, and Ag-4*d* in CO overlap near the −10 eV, −7.5 eV, and −2 eV energy levels, respectively, causing electron redistribution. Especially for the adsorption of C_2_H_4_, the atomic orbitals H-1*s* and C-2*p* of the gas molecules exhibit strong orbital hybridization with O-2*p* and Ag-4*d* in the energy level range of −7.5 eV to 2.5 eV, resulting in an elevation of the TDOS near the Fermi level.

**Figure 10:**
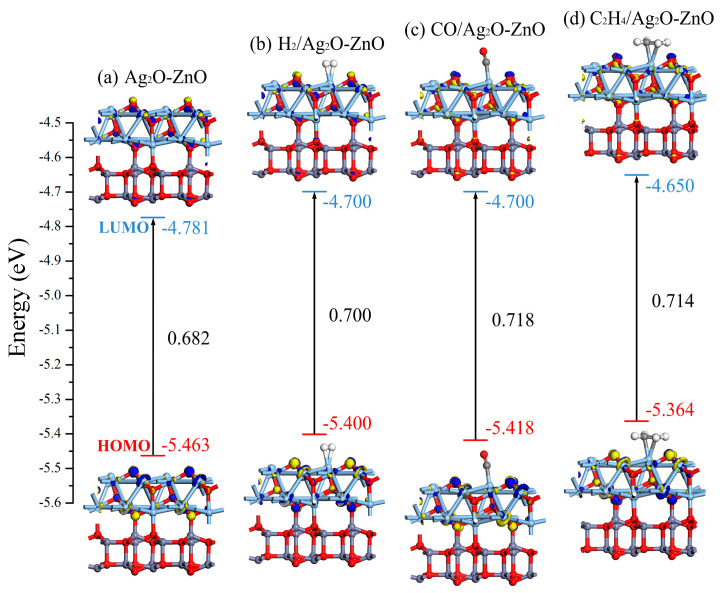
HOMO and LUMO distribution before and after gas adsorption on the Ag_2_O-ZnO heterojunction.

The distribution of HOMO and LUMO in heterojunctions and various adsorption systems were plotted and analyzed, and the values of *E_LUMO_*, *E_HOMO_*, and *E_g_* were calculated, as shown in Figure 10. It can be observed that among the three gas molecules, C_2_H_4_ adsorption had the greatest impact on the distribution of HOMO and LUMO in Ag_2_O-ZnO heterojunctions. Meanwhile, after adsorbing C_2_H_4_, the *E_LUMO_* of the system changed from −4.781 eV to −4.650 eV, *E_HOMO_* changed from −5.463 eV to −5.364 eV, and *E_g_* increased from 0.682 eV to 0.714 eV, indicating that C_2_H_4_ gas adsorption reduced the conductivity of the Ag_2_O-ZnO heterojunction. For the adsorption of H_2_ and CO, compared to the Ag_2_O-ZnO heterojunction, the *E_g_* values of these two adsorption systems also increased to 0.700 eV and 0.718 eV, respectively, resulting in a decrease in conductivity. The above molecular orbital analysis is consistent with the DOS analysis results mentioned earlier.

### 3.3. Gas Sensing Properties of the Cu_2_O-ZnO Heterojunction for H_2_, CO, and C_2_H_4_

#### 3.3.1. Structure and Electronic Analysis of the Cu_2_O-ZnO Heterojunction

**Figure 11:**
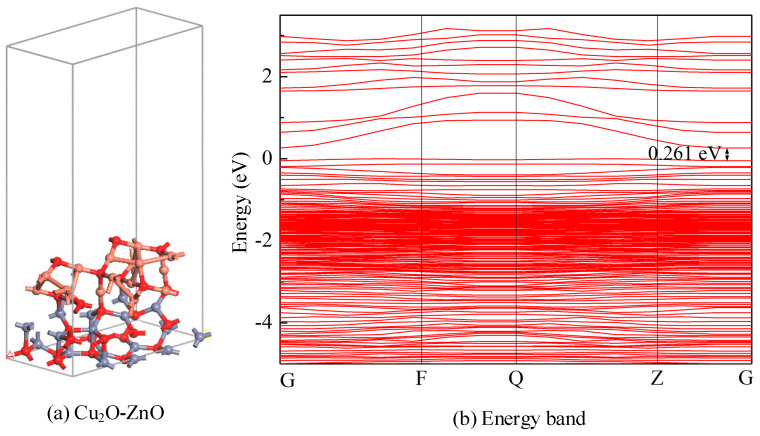
(**a**) Optimized structure of the Cu_2_O-ZnO heterojunction. (**b**) Energy band of the Cu_2_O-ZnO heterojunction.

A 2 × 1 × 1 Cu_2_O-ZnO two-layer heterojunction was constructed, where Cu_2_O serves as the upper layer metal oxide, while ZnO serves as the lower layer-based metal oxide. The lattice mismatch of the Cu_2_O-ZnO heterojunction is within a 5%. The structure of the Cu_2_O-ZnO metal oxide heterostructure is optimized as shown in Figure 11a, with lattice constants of *a* = 12.5367 Å, *b* = 6.2683 Å, and *c* = 29.9318 Å; *γ* = at 120.000°, containing 56 atoms. Meanwhile, by observing the geometric structure of the Cu_2_O-ZnO heterojunction, it can be observed that Zn-O and Cu-O bonds are formed between the two layers of metal oxides, with the longest bond length being 1.983 Å and the shortest being 1.905 Å. The difference in bond lengths is not significant and remains consistent. The calculated formation energy of the Cu_2_O-ZnO heterojunction model is −4.344 eV. The energy band of the optimized Cu_2_O-ZnO metal oxide heterojunction is shown in Figure 11b, with a bandgap width of 0.261 eV. After the formation of heterostructures, the band distribution is denser and the bandgap width is narrower compared to intrinsic Cu_2_O and ZnO. These phenomena reflect the improved electronic properties and increased carrier mobility of Cu_2_O-ZnO heterojunctions.

#### 3.3.2. Structure and DCD Analysis of the Gas/Cu_2_O-ZnO Heterojunction System

**Figure 12:**
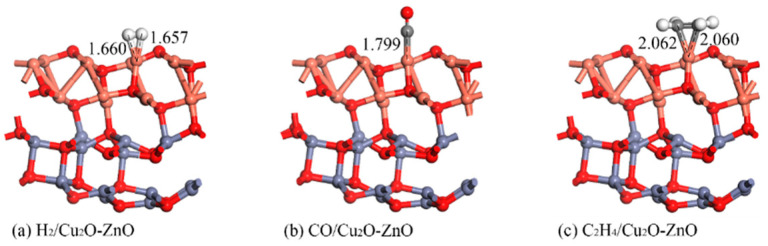
Structural models of H_2_, CO, and C_2_H_4_ adsorption on the Cu_2_O-ZnO heterojunction. The distance is in Å.

As in Section 3.1.2, the optimal adsorption models for the three gas molecules H_2_, CO, and C_2_H_4_ established on the surface of the Cu_2_O-ZnO heterojunction are shown in Figure 12, and the corresponding adsorption parameters are shown in Table 3. In the geometric optimization process involving molecular adsorption, the entire heterostructure was relaxed. For H_2_ adsorption, the calculated adsorption energy is −0.592 eV, which is slightly smaller than the adsorption on intrinsic ZnO, and much larger than the adsorption on intrinsic Cu_2_O. The two H atoms of H_2_ are close to the Cu atoms on the surface, forming two Cu-H bond lengths of 1.660 Å and 1.657 Å, respectively. For CO adsorption, the adsorption energy is −1.745 eV, which is higher than the that of gas adsorption on intrinsic ZnO and Cu_2_O. Meanwhile, the C atom is close to the Cu atom, forming a Cu-C bond with a bond length of 1.799 Å. For the adsorption of C_2_H_4_, the calculated adsorption energy is −1.649 eV, which is also higher than the adsorption energy of gas adsorption on intrinsic ZnO and Cu_2_O. The two C atoms of C_2_H_4_ are still close to the surface Cu atoms, forming two Cu-C bonds with bond lengths of 2.062 Å and 2.060 Å, respectively. Comparing the adsorption of three gas molecules on the heterojunction with the adsorption of two intrinsic metal oxides, ZnO and Cu_2_O, it is found that the adsorption distances of H_2_, CO, and C_2_H_4_ on the Cu_2_O-ZnO heterojunction shorten to a certain extent. Meanwhile, comparing the model diagrams before and after gas adsorption, it can be found that its structure has not changed significantly, indicating that the Cu_2_O-ZnO heterojunction has strong stability.

**Figure 13:**
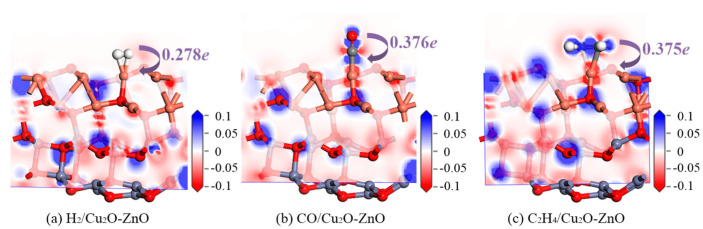
DCD diagrams of H_2_-, CO-, and C_2_H_4_-adsorbed Cu_2_O-ZnO heterojunctions.

Figure 13 shows the DCD distributions of H_2_, CO, and C_2_H_4_ adsorption on the surface of the Cu_2_O-ZnO heterojunction, with corresponding charge transfer values marked in the figure. As shown in Figure 13a, after H_2_ adsorbs on the surface of the heterojunction, a red color appears around the two H atoms, indicating a decrease in electron density in this region. Gas molecules transfer electrons to the surface of the heterojunction, and the transfer charge calculated based on charge distribution analysis is 0.278 *e*. Figure 13b shows the DCD distribution of CO adsorption on the surface. The O atom of the CO molecule is surrounded by a blue color, indicating the reception of foreign electrons, while the C atom appears to have a red color as a donor of electrons. Charge distribution analysis shows that the overall 0.376 *e* electrons transfer from CO gas molecules to the heterojunction’s surface. The DCD distribution of the C_2_H_4_ adsorption on Cu_2_O-ZnO heterojunction is shown in Figure 13c. The four H atoms of C_2_H_4_ are slightly reddish, resulting in a decrease in electron density. On the contrary, the two C atoms appear blue, indicating an increase in electron density in the region. Charge distribution analysis shows that C_2_H_4_ provides electrons to the heterojunction surface with a charge of 0.375 *e*. Similar to the previous discussion, in heterojunctions, O atoms always act as electron acceptors, while the metal atoms Zn and Cu always act as electron donors. Based on the structure and DCD analysis, it can be concluded that the adsorption of H_2_, CO, and C_2_H_4_ on the surface of the Cu_2_O-ZnO heterojunction is due to chemical adsorption. Especially for CO and C_2_H_4_ gas molecules, their interactions with the heterojunction surface are more intense.

#### 3.3.3. DOS and Molecular Orbital Analysis of the Gas/Cu_2_O-ZnO Heterojunction System

**Figure 14:**
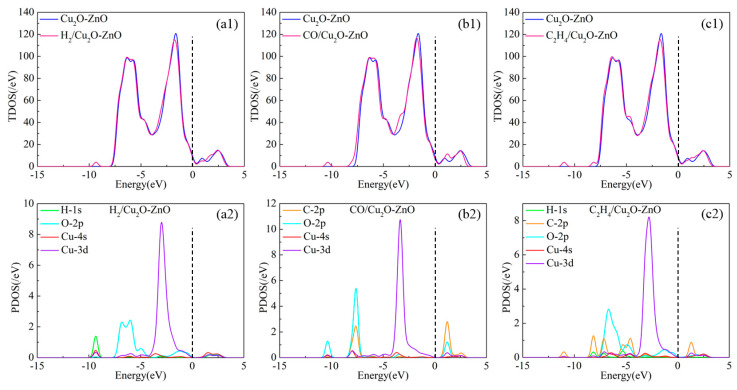
TDOS and PDOS of H_2_-, CO-, and C_2_H_4_-adsorbed Cu_2_O-ZnO heterojunctions. (**a1**) TDOS of H_2_-adsorbed Cu_2_O-ZnO heterojunction; (**a2**) PDOS of H_2_-adsorbed Cu_2_O-ZnO heterojunction; (**b1**) TDOS of CO-adsorbed Cu_2_O-ZnO heterojunction; (**b2**) PDOS of CO-adsorbed Cu_2_O-ZnO heterojunction; (**c1**) TDOS of C_2_H_4_-adsorbed Cu_2_O-ZnO heterojunction; (**c2**) PDOS of C_2_H_4_-adsorbed Cu_2_O-ZnO heterojunction.

The TDOS and PDOS of H_2_, CO, and C_2_H_4_ adsorption on Cu_2_O-ZnO heterojunction are shown in Figure 14. The position of the Fermi level is near the dotted line in Figure 14. By observing the TDOS before and after gas adsorption on the heterojunction, a comparative analysis shows that the TDOS of these three adsorption systems have varying degrees of increase near the Fermi level, indicating that the adsorption of gas molecules on the Cu_2_O-ZnO heterojunction leads to a decrease in its own conductivity. Specifically, the impact of atomic interactions on TDOS can be analyzed by observing PDOS. For H_2_ adsorption, H-1*s*, O-2*p*, and the atomic orbitals Cu-4*s* and Cu-3*d* of Cu atoms undergo orbital hybridization in the energy level ranges of −7.5 eV to 0 eV and 1 eV to 3 eV, respectively, which raises the TDOS curve near the Fermi level. For CO adsorption, the peaks of C-2*p* and O-2*p* of gas molecules overlap with those of the Cu-4*s* and Cu-3*d* of Cu atoms near the −7.7 eV, −3.5 eV, and 2.5 eV energy levels, while the peaks of C-2*p*, O-2*p*, and Cu-3*d* overlap near the 1.2 eV energy level, thereby affecting the distribution of TDOS. For the adsorption of C_2_H_4_, strong orbital hybridization occurs in the energy levels of H-1*s*, C-2*p*, O-2*p*, Cu-4*s*, and Cu-3*d* within the range of −8.7 eV to 0 eV and 1 eV to 3 eV, leading to electron redistribution and corresponding changes in the TDOS curve.

**Figure 15:**
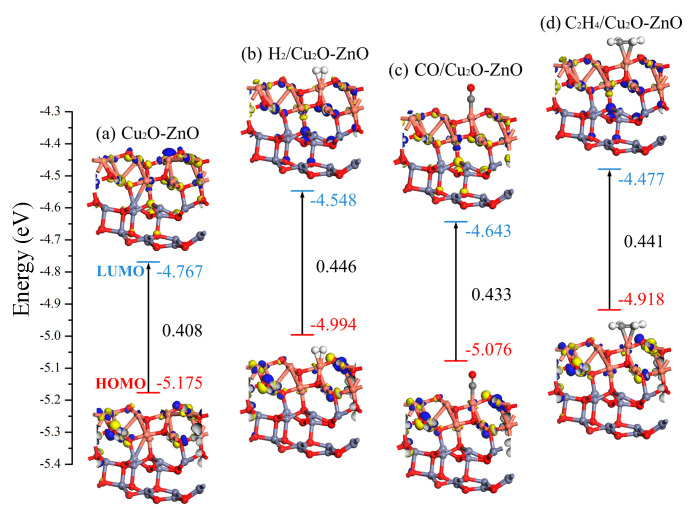
HOMO and LUMO distribution before and after gas adsorption on Cu_2_O-ZnO heterojunctions.

From the perspective of molecular orbital analysis, the influence of gas molecule adsorption on the electronic properties of heterojunctions was studied. The HOMO and LUMO distribution of the Cu_2_O-ZnO heterojunctions before and after gas adsorption were plotted, as shown in Figure 15. The corresponding values of *E_LUMO_*, *E_HOMO_*, and *E_g_* for each system were marked in the figure. It can be observed that the adsorption of H_2_, CO, and C_2_H_4_ has a significant impact on the HOMO and LUMO distribution of Cu_2_O-ZnO heterojunctions. In particular, the adsorption of C_2_H_4_ has a significant impact on the LUMO distribution of the Cu_2_O-ZnO heterojunction, and the corresponding *E_LUMO_* value increased from −4.767 eV to −4.477 eV. The *E_g_* value of the Cu_2_O-ZnO heterojunction is 0.408 eV, and the *E_g_* values of H_2_, CO, and C_2_H_4_ adsorbed systems increased to 0.446 eV, 0.433 eV, and 0.441 eV, respectively. Correspondingly, the conductivity of these three adsorption systems decreased. The above molecular orbital analysis is consistent with the DOS analysis results.

## 4. Conclusions

To track and monitor the running status and energy efficiency level of transformers in operation, this study proposes a TMO-ZnO (CuO-ZnO, Ag_2_O-ZnO, Cu_2_O-ZnO) heterojunction-based sensor for transformer dissolved gas (H_2_, CO, and C_2_H_4_) detection. The gas adsorption and sensing mechanism of the gas sensor to the gases were studied by analyzing the heterojunction model, adsorption structure, adsorption energy, adsorption distance, energy band, charge transfer, DOS, DCD, and molecular orbitals. This study explored the interaction mechanism between target gas molecules and TMO-ZnO heterojunction substrates. The main conclusions are as follows:

(1) The structures of TMO-ZnO (CuO-ZnO, Ag_2_O-ZnO, Cu_2_O-ZnO) heterojunctions were built and optimized. By observing the structure of the heterojunction model and the formation energies of the heterojunction structure, it can be found that the geometric structures of these three heterojunctions are stable enough. The analysis of the heterojunction energy bands showed that all three heterojunction energy bands had a certain degree of densification compared to the original metal oxides, and the bandgap width became narrower, indicating an improvement in the electrical conductivity of the system.

(2) The adsorption structures for H_2_, CO, and C_2_H_4_ on the TMO-ZnO (CuO-ZnO, Ag_2_O-ZnO, Cu_2_O-ZnO) heterojunctions were built and optimized. We analyzed and compared the adsorption energy, adsorption distance, and charge transfer of the adsorption system and conducted DOS and molecular orbital studies. The results indicated that the CuO-ZnO heterojunction has weak gas adsorption and sensing performance for H_2_, while it exhibits good gas sensing performance for CO and C_2_H_4_. Both Ag_2_O-ZnO and Cu_2_O-ZnO have significant gas sensing performance for H_2_, CO, and C_2_H_4_. The gas adsorption process does not destroy the original geometric configuration of the heterojunction, making TMO-ZnO heterojunctions suitable as gas sensing materials and as sensors for transformer defect detection and energy efficiency analysis.

## Figures and Tables

**Table 1:** Adsorption parameters of H_2_, CO, and C_2_H_4_ gas molecules on the CuO-ZnO heterojunction.

System	Distance (Å)	*E_ads_* (eV)	*Q_t_* (*e*)
H_2_/CuO	3.018	−0.160	0.008
CO/CuO	2.543	−0.280	0.070
C_2_H_4_/CuO	2.590	−0.578	0.159
H_2_/ZnO	2.938	−0.638	0.005
CO/ZnO	2.151	−0.770	0.234
C_2_H_4_/ZnO	2.426	−1.055	0.227
H_2_/CuO-ZnO	2.942	−0.161	0.011
CO/CuO-ZnO	2.480	−0.292	0.090
C_2_H_4_/CuO-ZnO	2.239	−0.922	0.326

**Table 2:** Adsorption parameters of H_2_, CO, and C_2_H_4_ gas molecules on the Ag_2_O-ZnO heterojunction.

System	Distance (Å)	*E_ads_* (eV)	*Q_t_* (*e*)
H_2_/Ag_2_O	2.116	−1.436	0.133
CO/Ag_2_O	2.042	−2.133	0.306
C_2_H_4_/Ag_2_O	2.337	−2.385	0.323
H_2_/Ag_2_O-ZnO	1.991	−0.346	0.160
CO/Ag_2_O-ZnO	2.026	−1.083	0.305
C_2_H_4_/Ag_2_O-ZnO	2.284	−1.300	0.295

**Table 3:** Adsorption parameters of H_2_, CO, and C_2_H_4_ gas molecules on the Cu_2_O-ZnO heterojunction.

System	Distance (Å)	*E_ads_* (eV)	*Q_t_* (*e*)
H_2_/Cu_2_O	2.678	−0.144	0.005
CO/Cu_2_O	1.809	−1.490	0.380
C_2_H_4_/Cu_2_O	2.070	−1.379	0.382
H_2_/Cu_2_O-ZnO	1.660	−0.592	0.278
CO/Cu_2_O-ZnO	1.799	−1.745	0.376
C_2_H_4_/Cu_2_O-ZnO	2.060	−1.649	0.375

## Data Availability

Data are contained within the article.
